# Unexpected Outcome of Daptomycin-induced Eosinophilic Pneumonia: Rarity Within a Rarity

**DOI:** 10.7759/cureus.6271

**Published:** 2019-12-02

**Authors:** Ahmad Raza, Ahmad Arslan, Muhammad Umair Atiq, Vincent Chan, Rajesh Kumar Patel

**Affiliations:** 1 Internal Medicine, Abington Hospital-Jefferson Health, Abington, USA; 2 Pulmonary and Critical Care Medicine, Abington Hospital-Jefferson Health, Abington, USA

**Keywords:** daptomycin, adverse effect, steroids, eosinophilic pneumonia

## Abstract

Eosinophilic pneumonia comprises a group of lung diseases in which eosinophils appear in increased numbers in the lungs and sometimes in the bloodstream. Among several causes of pulmonary eosinophilia, drug-induced pulmonary eosinophilia and subsequent pneumonia is a well-known side effect of many medications. Daptomycin is now being increasingly recognized culprit medication in patients with eosinophilic pneumonia. Here we present a patient with daptomycin-induced acute eosinophilic pneumonia, who had an unusual response to usual treatment.

## Introduction

Eosinophilic pneumonia is a rare but serious respiratory syndrome that occurs when eosinophils accumulate in the lungs [1]. The major causes of pulmonary eosinophilia include parasitic and fungal infections, medications, vasculitis, allergies, idiopathic eosinophilic pneumonia, and neoplasms. Among these, eosinophilic pneumonia has been associated with several drugs and chemicals, with antibiotics and nonsteroidal anti-inflammatory medicines among the most common [2]. The pathophysiology of acute eosinophilic pneumonia is thought to be caused by the detection of an antigen by alveolar macrophages, which lead to the recruitment of T-helper 2 lymphocytes and subsequent release of interleukin 5. Interleukin 5 promotes eosinophil production and migration to the lung. Additionally, eotaxin (a potent eosinophil chemoattractant) production by alveolar macrophages, pulmonary endothelial cells, airway smooth muscle cells, and alveolar epithelial cells leads to further accumulation of eosinophils in the lungs [3].

Daptomycin is a cyclic lipopeptide antibiotic derived from the fermentation of Streptomyces rose sports. Daptomycin has activity against Gram-positive organisms, including methicillin-resistant Staphylococcus aureus (MRSA) and vancomycin-resistant enterococci (VRE) [4]. In 2007, pulmonary eosinophilia was added to the “Adverse Reactions, Post-Marketing Experience” section of the product label for daptomycin. While the mechanism of daptomycin’s pulmonary toxicity is not known, the drug undergoes conformational change through interaction with calcium, which allows binding to the cytoplasmic membrane, increased membrane permeability, and intracellular ion escape [5].

Per the FDA guidance, eosinophilic pneumonia is attributed to daptomycin when the following criteria are met: 1) concurrent exposure to daptomycin, 2) fever, 3) dyspnea with increased oxygen requirement or requiring mechanical ventilation, 4) new infiltrates on chest X-ray or computed tomography (CT) scan, 5) bronchoalveolar lavage (BAL) with >25% eosinophils, and 6) clinical improvement following daptomycin withdrawal. Though not all the patients with suspected daptomycin-induced eosinophilia full all of the above criteria, it is still helpful in making a diagnosis.

Overall, dyspnea was the most common documented symptom associated with eosinophilic pneumonia, followed by the presence of either pulmonary infiltrates or opacities on chest X-ray or CT. Peripheral blood eosinophilia is, by no means, uniformly increased in all types of eosinophilic lung diseases [6, 7]. Corticosteroids are believed to be beneficial at halting clinical manifestations of daptomycin-induced eosinophilic pneumonia and were used in the majority of the previously reported cases. Steroids exert action through eosinophilic apoptosis and through accelerating intracellular signaling involved in eosinophil death [8].

## Case presentation

Our patient was a 72-year-old gentleman with the past medical history of mild chronic obstructive pulmonary disease (COPD), a previous history of subsegmental pulmonary embolism, atrial fibrillation status post watchman device, peripheral vascular disease status post right femoropopliteal bypass, hypertension, and hyperlipidemia. He was an ex-smoker with a 60 pack-year smoking history and quit about six years ago. He had no recent travel. His medications included rivaroxaban, aspirin, duoneb, metoprolol, amiodarone, and amlodipine. He was recently discharged from the hospital after being treated for right fifth toe osteomyelitis. He was treated with intravenous daptomycin because he had anaphylactic reaction to vancomycin in the past. He subsequently got this toe amputation done, but the proximal bone cultures grew MRSA, so he was continued on daptomycin post-operatively. An infectious disease specialist evaluated him, and he was discharged to rehab on a six-week course of daptomycin via a peripherally inserted central venous catheter (PICC).

**Figure 1:**
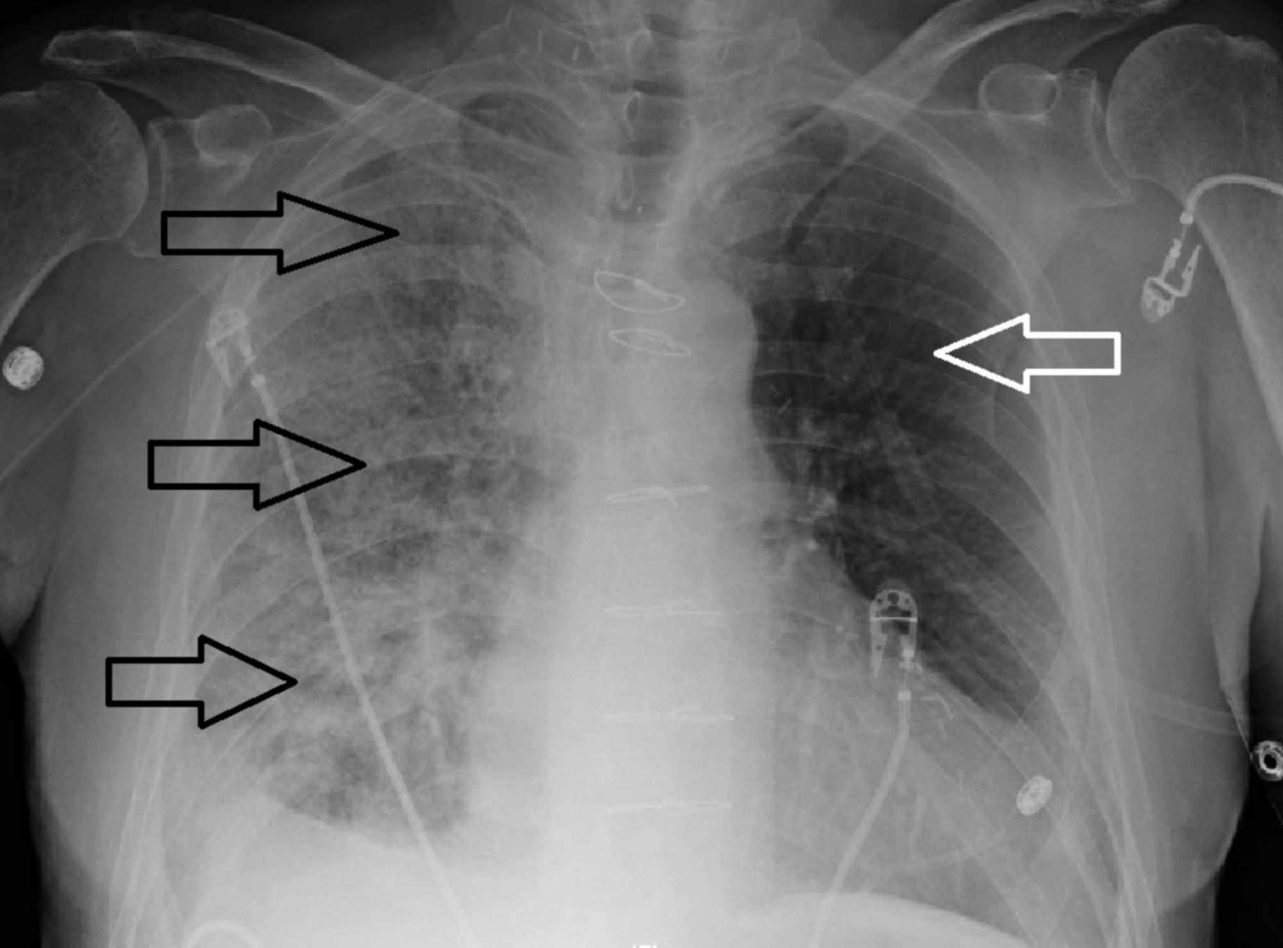
Portable chest X-ray demonstrating multifocal opacities involving all lobes of right lung (black arrows). Left lung parenchyma is relatively preserved (white arrow).

He was gradually recovering in rehab when his symptoms started with nausea along with non-bloody diarrhea of three days duration. He subsequently developed subacute respiratory distress and was requiring up to six liters of nasal cannula oxygen. He was sent to the hospital for further evaluation. In the emergency room (ER), he was found to be in mild respiratory distress, and his lungs exam showed bilateral crackles that were predominantly present on the right side. Heart exam was unremarkable except irregularly irregular pulse. Lower extremity exam showed well healing right lower extremity surgical scar without any edema. He was otherwise vitally stable on 4-6 liters of nasal cannula oxygen. His pertinent basic blood workup in the ER was as follows: Cr: 1.61 mg/dl, BUN: 20 mg/dl, cardiac BNP: 1432, INR: 1.7, Hb: 12.4 g/dl, WBC: 10.7 k/ul, absolute eosinophils count: 3.1 k/ul, absolute neutrophil count: 6.1 k/ul. His urinalysis was essentially unremarkable. He did not have a baseline cardiac BNP but had a normal baseline creatinine about four weeks back. In the ER, he was evaluated with a chest X-ray that showed new opacification throughout the right lung, suggestive of unilateral pulmonary edema or pneumonia (Figure 1).

He was started on linezolid and metronidazole for possible aspiration/healthcare-associated pneumonia along with intravenous (IV) diuresis to treat possible pulmonary edema. He was admitted on the general medical floor.

**Figure 2:**
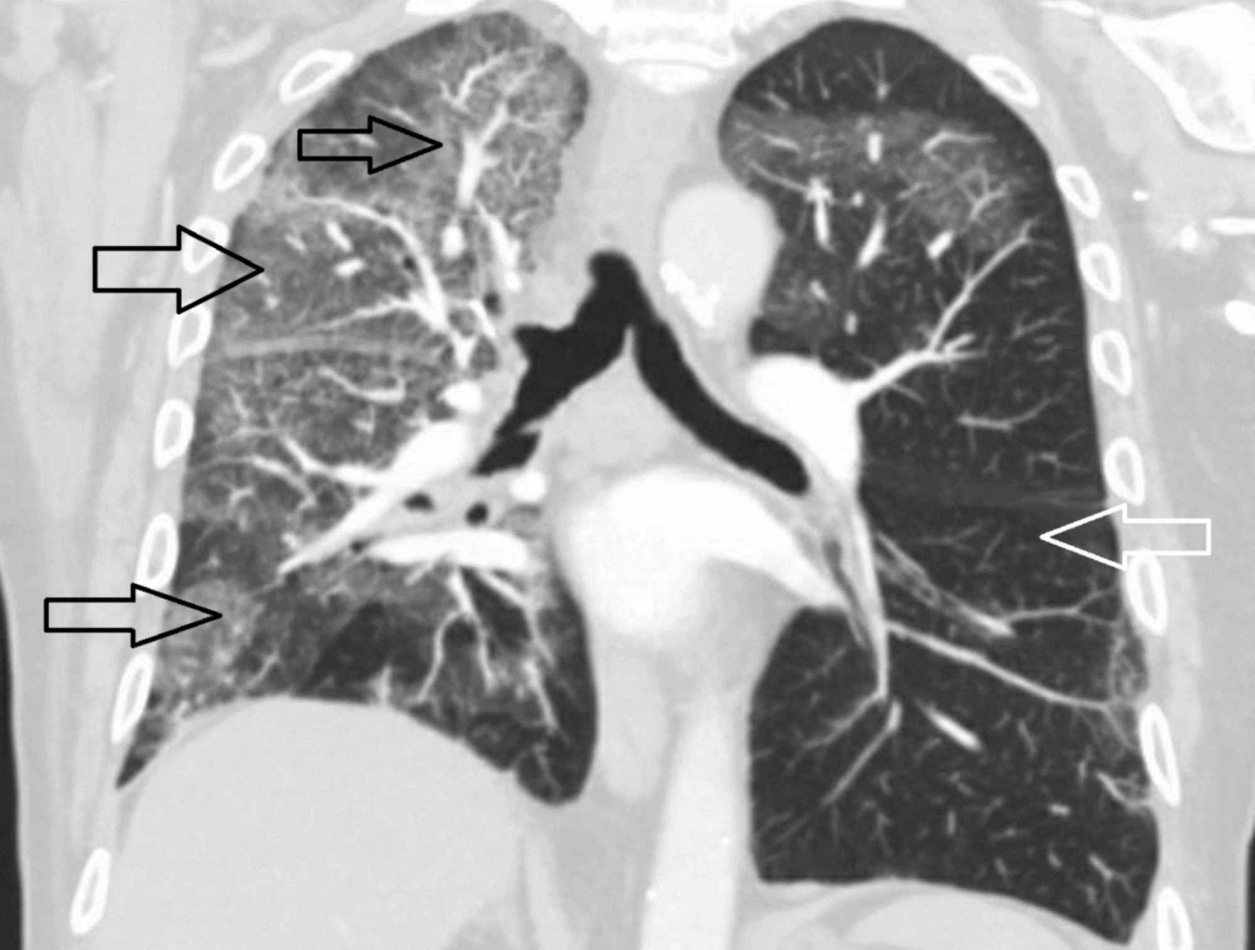
Coronal view of CT scan of thorax demonstrating extensive parenchymal lung disease involving all three lung lobes (black arrows). Left lung is relatively normal (white arrow).

**Figure 3:**
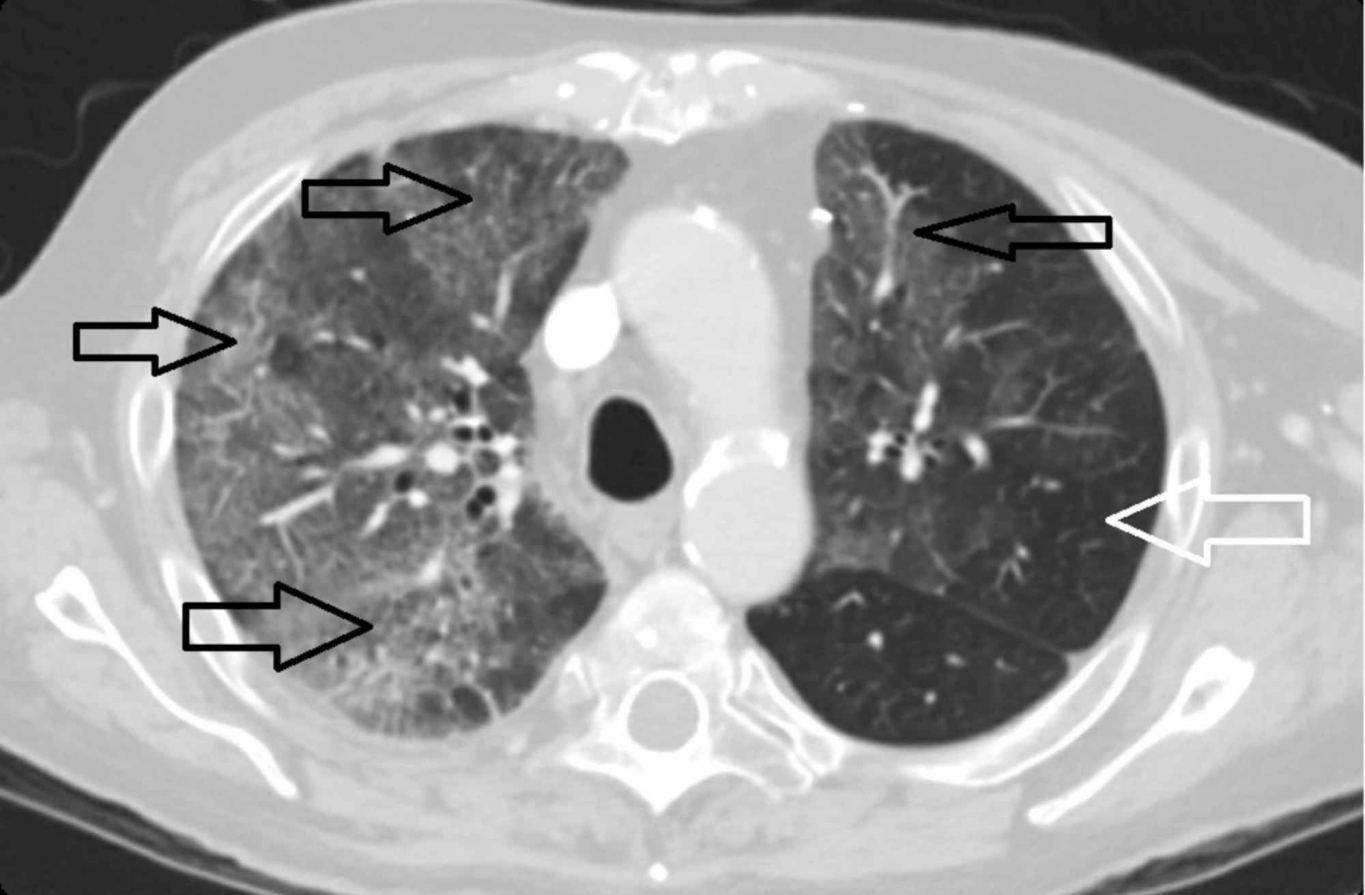
Cross section view of CT scan of thorax demonstrating bilateral right-sided predominant multi-focal parenchymal lung disease in the form of ground-glass opacities (black arrows). Left lung is less affected and shows areas of relatively preserved parenchyma (white arrow).

The next day we did a CT scan of his chest to evaluate the lung parenchyma better. The coronal view of CT scan of his chest showed bilateral ground-glass opacities of the lung parenchyma with right lung predominance (Figure 2). Similar findings of bilateral ground-glass opacities can also be seen on a cross-sectional view of the CT chest (Figure 3).

The echocardiogram of his heart showed normal systolic function without any evidence of diastolic dysfunction. He was continued on gentle intravenous diuresis, and the plan was to do a bronchoscopy to make a diagnosis. Meanwhile, we discontinued the daptomycin on admission. We continued to support his breathing with respiratory treatments with albuterol/ipratropium, supplemental oxygen for hypoxia, antibiotics for possible bacterial pneumonia, and diuretics for possible pulmonary edema. Bronchoscopy was done after elective intubation, and gross inspection only showed clear secretions throughout the right lung. Bronchoalveolar lavage was done from the right upper/middle lobe, and fluid was sent for bacterial/fungal cultures/cytology/AFB smear and differential count. After the bronchoscopy, the patient was started on prednisone 40 mg IV twice a day. His bronchoscopic fluid analysis showed a total WBC count of 8504/UL, with 40% neutrophils and 42% eosinophils. Bacterial/fungal cultures were negative for infection, and cytology was negative for malignant cells.

At this point, based on the clinical presentation and above lab data, a diagnosis of daptomycin-induced eosinophilic pneumonia (DAP) was made. He was extubated and was transferred to the general medical floor on nasal cannula oxygen. Antibiotics and IV diuretics were stopped, and he was started on Trimethoprim-Sulfamethoxazole for pneumocystis (PCP) prophylaxis in anticipation of long-term steroid treatment. His oxygen requirement varied from six liters of nasal cannula oxygen to a 55% venturi mask for the next few days. Recovery, in his case, was relatively slow despite being on steroids, and he continued to require more than 40% FiO2 for more than a week. Given his slow recovery and ongoing relatively high oxygen requirement, he was started back on IV diuretics. He was evaluated by the cardiologist to assess his volume status better, and it was decided to do a right heart catheterization. Though the suspicion was low, we decided to stop his amiodarone as a potential contributor towards his ongoing pneumonitis. Right heart catheterization was mostly unremarkable and showed pulmonary capillary wedge pressure (PCWP) of 5. Diuretics were stopped at this point, and the patient was clinically observed on steroids. He had a prolonged recovery with possible residual lung damage as we were not able to entirely wean him off of oxygen despite two weeks of steroids. Though he was stable on 2-3 liters of nasal cannula oxygen at baseline, he was not on oxygen, and therefore it was new for the patient. He was subsequently discharged from the hospital in a stable condition and on home oxygen, with a close follow-up with the pulmonologist as an outpatient. Lung biopsy was never performed on this patient.

## Discussion

Acute eosinophilic pneumonia (AEP) is a subtype of acute pneumonia which is idiopathic or induced by drugs or toxins. It is distinct from atopic disease and autoimmune, parasite, or fungal infections that can also cause pulmonary eosinophilia. Among medications causing AEP, daptomycin is now being increasingly recognized and reported. Daptomycin is usually used against MRSA as a second-line agent after vancomycin in treating skin, soft tissue infections, and bacteremia. Early after the approval of daptomycin by the FDA, multiple cases of daptomycin-induced eosinophilic pneumonia have been reported. Hayes Jr. et al. reported the first case of daptomycin-induced eosinophilic pneumonia in 2007 [5]. Since that case report, more and more cases have been reported of a similar sort.

Based on the criteria, our patient had a diagnosis of daptomycin-induced eosinophilic pneumonia. Diagnosis of daptomycin-induced AEP is based on clinical history, laboratory results, and radiographic findings. Usually, patients present with dyspnea and cough with or without fever. The clinical presentation may vary from mild respiratory complains to minimal requirement of oxygen, up to acute respiratory distress syndrome (ARDS). Laboratory workup may reveal peripheral eosinophilia, leukocytosis, or elevated markers of inflammation such as erythrocyte sediment rate (ESR) and C-reactive protein (CRP). A chest X-ray reveals bilateral pulmonary infiltrates. Chest CT may reveal bilateral consolidations, infiltrates, pulmonary nodules. In our patient, chest X-ray showed predominantly right lung disease for unknown reasons. Though the disease was multi-lobular and bilateral, the predilection for the right lung in our patient was unusual and never documented before. Identification of the specific drug associated with the pneumonia is difficult because rechallenge with the drug is neither warranted nor safe. Dosage adjustment or therapeutic drug monitoring of DAP is not generally necessary; however, another study indicated that the pharmacodynamics of DAP vary markedly under different circumstances, such as in patients with morbid obesity, severe sepsis, or varying degrees of acute kidney injury [9].

In idiopathic AEP, most of the time, the peripheral eosinophil count is not elevated in the early phases but may become elevated later on during the course of the disease [10]. Our patient did have eosinophilia. Peripheral eosinophilia is not necessary to diagnose AEP, and it is not part of the current diagnostic criteria, but it may suggest eosinophilic pneumonia [11]. Recently, a normal eosinophil count at the initial presentation was reported to be more common in smoking-related AEP than in idiopathic and medication-induced AEP. This observation is consistent with the data from smoking-challenge tests demonstrating the initial development of blood and BAL neutrophilia after acute cigarette smoke exposure [11]. Other laboratory features include elevated erythrocyte sedimentation rate and C-reactive protein level. Serum IgE levels, when measured, may be moderately elevated but are not useful for the diagnosis of AEP [12].

The mainstay of daptomycin-induced AEP and any other drug-induced AEP is to give corticosteroids and to stop the offending drug. This practice is based on previous case reports of daptomycin-induced AEP and other medication-induced lung injuries. Steroids are recommended to patients with acute lung injuries caused by amiodarone, cocaine, and bleomycin. Most of the case reports on daptomycin-induced AEP responded almost immediately within 48-72 hours with dramatic improvement in patient’s symptoms [13]. In our patient, while the diagnosis was supported by clinical criteria, radiographic data, peripheral eosinophilia, history of drug use, and very high eosinophil count of BAL fluid, we struggled with his management despite starting the patient on steroids and optimizing the fluid status. He was still on up to 3 liters nasal cannula oxygen despite being in the hospital and being on steroids for about two weeks. We attributed his slow recovery to baseline emphysematous lung disease and to the fact that he was concomitantly on amiodarone for his atrial fibrillation, which in itself can cause significant lung toxicity. His amiodarone was discontinued during hospitalization. Additive pulmonary side effects of these medications need to be further studied and elucidated, especially in regards to daptomycin.

## Conclusions

Daptomycin-induced AEP is a relatively new and rare side effect. Physicians should be aware of daptomycin-induced AEP in any patient who is actively on daptomycin and presenting with respiratory complaints. Daptomycin-induced AEP is not related to drug dose, and timing of presentation is reported to be very variable. Our case highlights a unique and rare side effect of daptomycin and raises further questions regarding addictive drug toxicities. Daptomycin-induced AEP can have delayed and incomplete recovery in patients with baseline lung diseases, and is another area of future research.
